# Enhanced Gender Recognition System Using an Improved Histogram of Oriented Gradient (HOG) Feature from Quality Assessment of Visible Light and Thermal Images of the Human Body

**DOI:** 10.3390/s16071134

**Published:** 2016-07-21

**Authors:** Dat Tien Nguyen, Kang Ryoung Park

**Affiliations:** Division of Electronics and Electrical Engineering, Dongguk University, 30 Pildong-ro 1-gil, Jung-gu, Seoul 100-715, Korea; nguyentiendat@dongguk.edu

**Keywords:** image quality assessment, gender recognition, visible light camera image, thermal camera image

## Abstract

With higher demand from users, surveillance systems are currently being designed to provide more information about the observed scene, such as the appearance of objects, types of objects, and other information extracted from detected objects. Although the recognition of gender of an observed human can be easily performed using human perception, it remains a difficult task when using computer vision system images. In this paper, we propose a new human gender recognition method that can be applied to surveillance systems based on quality assessment of human areas in visible light and thermal camera images. Our research is novel in the following two ways: First, we utilize the combination of visible light and thermal images of the human body for a recognition task based on quality assessment. We propose a quality measurement method to assess the quality of image regions so as to remove the effects of background regions in the recognition system. Second, by combining the features extracted using the histogram of oriented gradient (HOG) method and the measured qualities of image regions, we form a new image features, called the weighted HOG (wHOG), which is used for efficient gender recognition. Experimental results show that our method produces more accurate estimation results than the state-of-the-art recognition method that uses human body images.

## 1. Introduction

Currently, digital biometrics systems have been developed to provide many convenient features. For example, people can use their fingerprints and/or finger-vein patterns as a key to their house, passwords for logging in to some digital systems (computers, networks, automated teller machines (ATMs) at a bank), etc. [1,2]. Face and/or iris patterns can be used to identify individuals and detect criminals at the airport [1,3]. The gender (male/female) of a person can be important information. This kind of information is used in many biometric systems such as surveillance systems, age estimation systems, and face recognition systems. Recently, surveillance systems have been widely applied in public areas such as at airports, shopping malls, and libraries. These systems provide services for monitoring and controlling public areas. For surveillance systems, the extraction of gender information from observed persons is important for performing several tasks. In a shopping mall, the distribution of the gender of customers who buy specific products is important information for the shop owner in their development plan. In addition, the shop owner can show different advertisements based on the gender of customers (male or female) who stand in front of an advertisement panel. In some public areas, knowing gender can help the manager create restricted male and female areas [4]. 

The human body contains information about the gender of each person in several locations such as the face, fingerprints, and body gait. Gender can often be observed using human perception. However, gender recognition is still a challenge for computer vision systems. Many previous studies to recognize gender using several features of the human body have been conducted [5,6,7,8,9,10,11,12,13,14,15,16]. Most previous research focused on gender recognition using the human face [5,6,7,8,9]. Using visualization, we can recognize gender using face images. Face-based gender recognition methods extract the features of a face and classify the face images into male or female. Through many previous studies, this kind of recognition method produces very high recognition accuracy. However, this kind of method requires the subject to be a short-distance from camera and to be cooperative; therefore, they are difficult to apply to surveillance systems.

To the best of our knowledge, there is very little previous research on gender recognition method using human body images. For building surveillance system applications, several studies on computer vision use body gait to estimate gender [10,11]. Males and females tends to walk with different gaits. Based on this assumption, these methods accumulate gait information from a sequence of images to recognize gender. In some other methods, the 3-D body shape of a human body was used for gender recognition [12,13]. First, the 3-D body shape of a human is captured using a laser scanner. Then, several specific characteristics in the 3-D models are analyzed to discriminate between males and females. Although these methods produce good recognition results, they require the cooperation of subjects during image acquisition. In addition, capturing a 3-D image using a laser scanner is more expensive than using normal cameras.

To increase user convenience, previous studies used visible light images of a human body for gender recognition [14,15]. The research by Cao et al., showed that visible light images of the human body can be used for reliable gender recognition [14]. By performing experiments on a public visible light database (the MIT pedestrian database), they showed that the recognition accuracy of their method, based on a histogram of oriented gradient (HOG) and a boosting method, was roughly 75%. Later, work by Guo et al. [15] enhanced recognition accuracy to roughly 80% using biologically-inspired features (BIFs) and a machine learning method on the same database (the MIT pedestrian database). These researches have a limitation of using single visible light images for gender recognition system. Therefore, the recognition error is still high. Most recently, Nguyen et al. proposed a method for enhancing the recognition accuracy of a gender recognition system using images of the human body [16]. In this method, they combined the appearance of human body in two types of images, including visible light and thermal images. In addition, the recognition performances of recognition system using two difference feature extraction methods of HOG and multi-level local binary pattern (MLBP) were shown and the HOG feature outperforms the MLBP feature for gender recognition problem. By exploiting gender information from two different kinds of images, the recognition accuracy was enhanced to roughly 85%, which is higher than that when using single visible light images (as in previous research). However, they did not consider the quality assessment of visible light and thermal images of human body.

Although the use of images of the human body offers several advantages, i.e., it does not require the cooperation of subjects, is easy to implement, and is suitable for surveillance systems, the performance of this kind of recognition method is strongly affected by the background regions and the large variation in human body texture (due to clothes, accessories, etc.). Whereas the variation of human body texture is very difficult to address because it depends on individuals and appears to be random, the effects of background regions can be reduced by employing thermal images. In previous research, the effects of the background are difficult to reduce using only single visible light images. Because the thermal images capture the infrared light that is radiated from the human body based on its high temperature as compared to background regions, human body regions are normally displayed brighter than background regions on thermal images. Based on this characteristic, we propose a new method for gender recognition based on the combination of visible light and thermal images, and quality assessment of the image’s regions, so as to reduce the effect of background regions on recognition system. In Table 1, we show a summary of previous research on gender recognition and the strengths and weaknesses of each method in comparison with our proposed method.

The remainder of our paper is as follows: in Section 2, we propose a new method for gender recognition using the combination of visible light and thermal images, and a quality assessment method, so as to reduce the effects of the background and noise, and enhance the recognition accuracy. Section 3 shows the experimental results when using our proposed method in a surveillance system application. Finally, our conclusions are presented in Section 4.

## 2. Proposed Method

### 2.1. Overview of the Proposed Method

**Figure 1:**
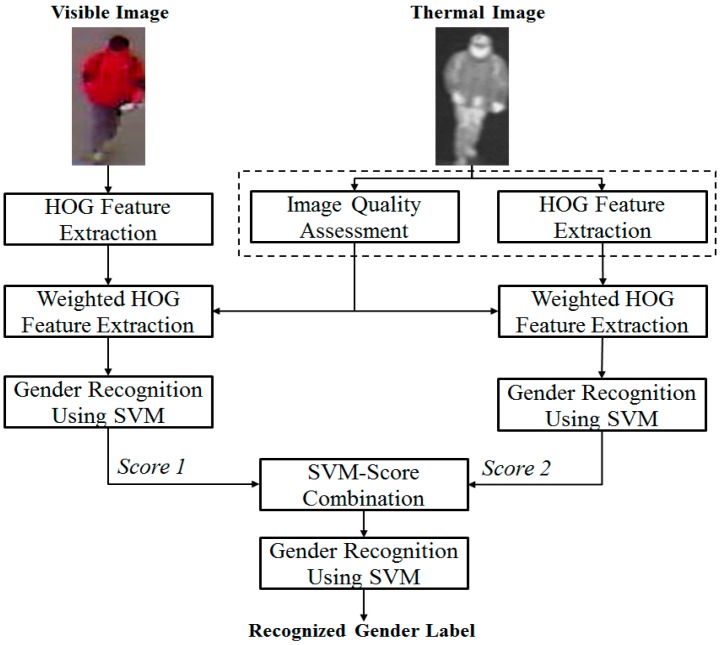
Overall procedure of our proposed method for gender recognition using visible light and thermal images, including image quality assessment.

In Figure 1, we present the overall procedure of our proposed method for gender recognition using a combination of visible light and thermal images, and a quality assessment method of image regions using the thermal image.

As shown by previous research by Nguyen et al., the combination of visible light and thermal images is better than the use of either visible light images or thermal images for gender recognition purposes [16]. Based on this result, we use both visible light and thermal images of the human body to perform gender recognition. As shown in Figure 1, the inputs of our system are images of the human body, including visible light images and thermal images. For the visible light images, the human body image is inputted into the HOG feature extraction method. For thermal images, we assess the quality of sub-blocks (image regions), in addition to extracting image features using the HOG method as in the visible light images. By combining the extracted HOG features with the quality measurement of image regions, our method forms a new feature, called the wHOG feature, which will be used to perform gender recognition. A detailed explanation of feature extraction using the HOG method will be presented in Section 2.2.1. In addition, we will explain the details of the quality assessment method of image regions using thermal images in Section 2.2.2. With the new extracted features, i.e., wHOG features, our method uses a support vector machine (SVM) to recognize gender. As reported in previous research by Nguyen et al., there are several methods for combining visible light and thermal images, such as feature-level fusion or score-level fusion, using linear or RBF kernels of the SVM [16]. Among these combination methods, score-level fusion using the RBF kernel was proven to work better than other methods for gender recognition. Therefore, our proposed method also uses score-level fusion using the RBF kernel for combining the visible light and thermal images. For this purpose, the extracted wHOG features from visible light and thermal images are inputted to the first SVM layer to recognize gender using a single kind of image. For each kind of image, we obtain a score (a decision value) that indicates the probability of the input image belonging to the male or female class. By combining the two scores produced by the visible light and thermal images, we recognize gender using a second SVM layer. Although the previous research claims that the score-level fusion approach using the RBF kernel of the SVM works better than other methods, we will use all of the above-described methods (feature-level fusion and score-level fusion with either a linear kernel or an RBF kernel) for combining the visible light and thermal images, compare the recognition results to those of previous methods, and confirm that score-level fusion using the RBF kernel is superior. 

### 2.2. Feature Extraction Method for Gender Recognition

In our proposed method, we use both visible light and thermal images of a person for gender recognition problem. The input images captured by visible light and thermal cameras contain the random texture and objects of the observed scene. In gender recognition problem, person detection and localization must be performed before any further processing steps. Similar to our previous research [16], we use the pedestrian detection method proposed by Lee et al., to detect and localize persons in both visible light and thermal images [17]. By employing both visible light and thermal images, Lee et al. show the method of detecting persons in various environments. In addition, the person can be detected in both images with small miss-alignments by employing the calibration method.

#### 2.2.1. Histogram of Oriented Gradient

**Figure 2:**
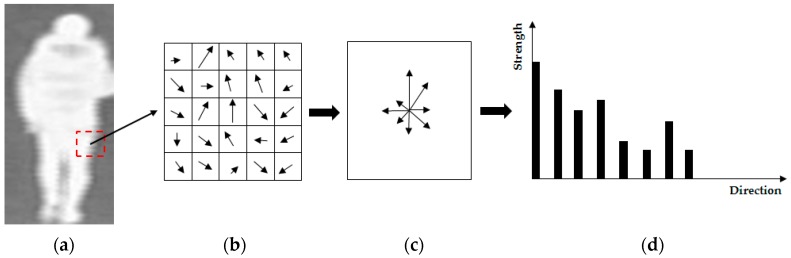
A demostration of the HOG feature extraction method: (**a**) the input image; (**b**) gradient map with gradient strength and direction of a sub-block of the input image; (**c**) accumulated gradient orientation; and (**d**) histogram of oriented gradients.

To extract the image features for gender recognition, our proposed method uses the HOG method. As shown in previous researches by Cao et al. [14] and Nguyen et al. [16], the HOG feature has been used for gender recognition. Originally, the HOG feature was successfully used for pedestrian detection [18,19,20]. Later, this feature extraction method was also successfully used for gender recognition [14,16], age estimation [21], and face recognition [22,23]. The reason the HOG feature works well for pedestrian detection and gender recognition is that the HOG feature measures the gradient information (strength and direction) of texture features in local regions of images. Therefore, by concatenating the HOG features of all local regions (sub-blocks) in an image, the extracted HOG feature accurately describes the shape and gait of a human body. In Figure 2, we present a demonstration of the HOG feature extraction methodology. In this implementation, the gradient maps in the vertical and horizontal directions are first calculated using vertial and horizontal gradient masks, respectively. To capture the local gradient information, the input image is then divided into several overlapped sub-blocks in both vertical and horizontal directions. Based on the vertical and horizontal gradient images, the directions of the image’s features at every pixel are then calculated, as shown in Figure 2b. In each sub-block, a histogram feature is accumulated based on the gradient strength and the direction of every pixel in the sub-block (Figure 2b–d).

#### 2.2.2. Image Quality Assessment of Local Regions

**Figure 3:**
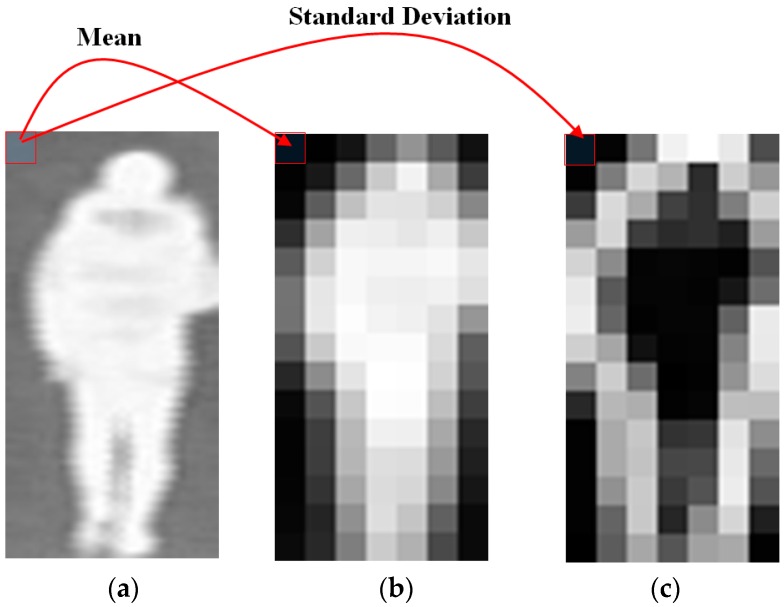
Example of mean and standard deviation maps obtained from a thermal image: (**a**) a thermal image with background (low illumination regions) and foreground (high illumination regions); (**b**) MEAN map; and (**c**) STD map.

In Figure 1 and Figure 2, we showed several examples of thermal images of the human body. We observe that the thermal images have the special characteristic of brightness (illumination level) of body regions and background regions. As mentioned in Section 1, the thermal images are obtained by capturing the infrared light that radiates from the observed scene because of the object’s temperature. Normally, the human body and the background regions have different temperature. Whereas the background’s temperature mostly depends on the environment temperature, the human body has a constant of temperature of roughly 37 °C. In normal cases, this temperature of the human body is higher than that of the environment; therefore, the human body will be displayed brighter than the background in the thermal image, as shown in Figure 1, Figure 2 and Figure 3. This characteristic not only helps our system to detect the human region in the low illumination condition (at night), but also offers a method to reduce the effects of the background region on recognition accuracy.

Our proposed method uses the HOG feature extraction method to extract the image’s features. As we described in Section 2.2.1, the HOG method extracts the strength and direction of texture features in local regions (sub-blocks) of the input image. We can easily observe that the background regions contain no gender information. Consequently, the sub-blocks that belong to the background regions should be removed from the extracted features. Based on this observation, we propose a quality assessment method of image regions so as to reduce the effects of the background on the recognition accuracy using the characteristics of thermal images. In detail, our proposed method will assign a very small weight value (close to zero) to background regions and will assign a larger weight value to foreground regions (regions that belong to the human body region). Based on the characteristics of thermal images, we propose using the mean and standard deviation of the gray levels of the sub-blocks of thermal images to measure the quality of sub-blocks in visible light and thermal image (called the MEAN map and STD map, respectively). 

As mentioned above, the foreground region (i.e., the human body region) is normally brighter than background regions. Therefore, if the mean of a sub-block (image regions) is small, there is a high probability that the sub-block belongs to the background region. In contrast, if the mean of a sub-block is high, the sub-block has a high probability of belonging to the human body region. For sub-blocks that contain both background and foreground regions (boundary sub-blocks), the mean values are medium (higher than the mean of the background sub-blocks and lower than the mean of the foreground sub-blocks). The mean map is used to measure the probability that a sub-block belongs to the background or the foreground; our proposed method also uses the standard deviation map (STD map) to indicate the probability of a sub-block containing the boundaries of the human body. As shown in Figure 1 and Figure 2, the human body regions are displayed brighter than the background regions in thermal images. In addition, the sub-blocks that completely belong to background or foreground have similar gray levels. Consequently, the standard deviation of sub-blocks that completely belong to background and foreground regions are much smaller than those at the human body boundaries, because of the transition of brightness from background (low brightness) to foreground regions (high brightness). In our method, we suppose that the background regions and the plain texture on the human body regions have less gender information than the other regions. Using the standard deviation values of sub-blocks, our proposed method can remove the effects of plain textures on the extracted features. In Figure 3 we show an example of the mean map (Figure 3b) and the standard deviation map (Figure 3c) obtained from a thermal image (Figure 3a). In Figure 3b,c, the brighter sub-blocks indicate higher quality values of sub-blocks. 

Figure 3b shows that the mean map can describe the foreground regions by assigning high weight values to foreground sub-blocks and small weight values to background sub-blocks. In Figure 3c, the standard deviation map assigns high weight values to boundary sub-blocks and assigns low weight values to both background regions and the plain texture regions. To measure the quality of sub-blocks, the calculated mean and standard deviation of a sub-block is normalized in the range of 0 to 1 using a min-max scaling method using all of the mean or standard deviation values of an image, respectively. We denote the quality measure using a mean value as MEAN, and the quality measure using standard deviation values as STD. Therefore, Equations (1) and (2) are used to create a balance of quality measurements of sub-blocks in an image:

(1) $$\text{MEAN}=\left\{m_{11},m_{12},\ldots ,m_{pq}\right\}\;where\;\overset{p}{\underset{i=1}{\sum }}\overset{q}{\underset{j=1}{\sum }}m_{ij}=1.0$$

(2) $$\text{STD}=\left\{std_{11},std_{12},\ldots ,std_{pq}\right\}\;where\;\overset{p}{\underset{i=1}{\sum }}\overset{q}{\underset{j=1}{\sum }}std_{ij}=1.0$$

In these equations, *m_ij_* and *std_ij_* are the mean and standard deviation values of sub-block in the *i*-th (horizontal) and *j*-th (vertical) positions, respectively. *p* and *q* are the number of sub-blocks in the horizontal and vertical directions, respectively. Because images of the human body in visible light and thermal images are simultaneously detected using the method by Lee et al., the misalignment between two images is very small. Therefore, our proposed method supposes that human body regions in visible light and thermal images are overlapped. Consequently, the quality measure of a sub-block in the thermal images can be used as the quality of the corresponding sub-block in the visible light image. In our experiments, we will use the mean map and standard deviation map as the quality measurement of sub-blocks of input images for both visible light and thermal images. Through experimental results, the mean map is efficiently used as the quality measurement for visible light images, whereas the standard deviation map is efficiently used as the quality measurement for thermal images. 

#### 2.2.3. Feature Combination and Gender Recognition Using SVM

**Figure 4:**
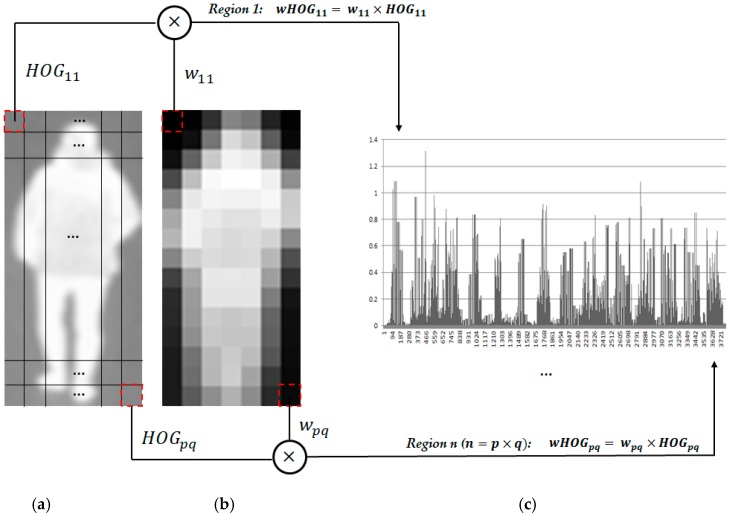
Demonstration of methodology for extracting the wHOG feature by combining the HOG features of images and weighted values of corresponding sub-blocks: (**a**) input image; (**b**) quality measurement map (MEAN map or STD map); and (**c**) the wHOG feature by combining (**a**) and (**b**).

Using the quality measurements of sub-blocks obtained using the MEAN map or the STD map of the thermal image, our proposed method constructs the novel wHOG feature of an image by combining the quality values and the corresponding HOG features of sub-blocks. If we call the quality measurement of sub-blocks of an image (the MEAN map in Equation (1) or the STD map in Equation (2)) $Q=\left\{w_{11},\ldots ,w_{ij},\ldots ,w_{pq}\right\}$ and the extracted HOG features from this image are $\text{HOG}=\left(HOG_{11},\;\ldots ,HOG_{ij},\ldots ,HOG_{pq}\right)$, where the *p* and *q* are the number of sub-blocks of the input image in the horizontal and vertical directions, respectively; *HOG_ij_* indicates the HOG feature (histogram feature) extracted from the sub-block at the *i*-th and *j*-th positions in the horizontal and vertical directions. The weighted HOG feature is formed using Equation (3) by multiplying the HOG features of a sub-block by the corresponding weight value of those sub-blocks. In this equation, *i* and *j* are the index values; they satisfy the conditions 0<i≤p and 0<j≤q. To clarify this concept, we present an example of methodology for making weighted HOG feature using Equation (3) in Figure 4. In this figure, the weighted HOG feature of a thermal image is constructed by modifying the HOG features of every sub-block of the image using the quality measurements of the corresponding sub-blocks based on the MEAN map:

(3) $$\text{wHOG}=\left(w_{ij}\times HOG_{ij}\right)=\left(w_{11}\times HOG_{11},\;w_{12}\times HOG_{12},\ldots ,w_{pq}\times HOG_{pq}\right)$$

As we explained in Section 2.2.2, we have two methods for quality measurement of image regions, including the MEAN map and the STD map. In our research, we consider three methods for constructing the wHOG features, including using the MEAN map as the quality measurement of sub-blocks for both visible light and thermal images (Method 1), using the STD map as the quality measurement of sub-blocks for both visible light and thermal images (Method 2), and using the MEAN map as the quality measurement of sub-blocks for visible light images and the STD map as the quality measurement of sub-blocks for thermal images (Method 3). The reason for exploiting Method 3 in our search will be explained in Section 3, showing the experiment results in which the MEAN map is suitable for enhancing the recognition result using the visible light image, whereas the STD map is suitable for enhancing the recognition result using thermal images.

In the first method, we use the MEAN map for constructing the weighted HOG feature. Consequently, Equation (3) changes to Equation (4) for the visible light image and Equation (5) for thermal images. In these equations, *MEAN_ij_* indicates the mean value of the sub-block’s *i*-th and *j*-th positions in the horizontal and vertical directions, respectively. *vHOG_ij_* and *tHOG_ij_* indicate the HOG features of visible light and thermal images obtained from sub-block at *i*-th and *j*-th positions in horizontal and vertical directions, respectively. Using the MEAN map for quality measurement of image regions, we can capture gender information in regions inside the body part and the body’s boundary parts and remove the information of background regions:

(4) $${\text{wHOG}}_{visible}=\left(MEAN_{ij}\times vHOG_{ij}\right)$$

(5) $${\text{wHOG}}_{thermal}=\left(MEAN_{ij}\times tHOG_{ij}\right)$$

In the second method (Method 2), the MEAN map is replaced by the STD map in Equations (4) and (5) to construct the wHOG feature for both visible light and thermal images. Consequently, the wHOG feature for visible light and thermal images are constructed using Equations (6) and (7) for this method. In these equations, *STD_ij_* indicates the standard deviation of the sub-block at the *i*-th and *j*-th positions in horizontal and vertical directions, respectively. Using the STD map, the wHOG feature can capture gender information at the boundaries of the body and remove information of background regions and inside-body regions where the plain texture features appear because of the similar temperature of inside-body regions:

(6) $${\text{wHOG}}_{visible}=\left(STD_{ij}\times vHOG_{ij}\right)$$

(7) $${\text{wHOG}}_{thermal}=\left(STD_{ij}\times tHOG_{ij}\right)$$

Using Methods 1 and 2, we can assess the recognition ability using each map of MEAN map (Method 1) and STD map (Method 2). As we will show later in our experimental results section (Section 3), the MEAN map is suitable for gender recognition using visible light images, and the STD map is suitable for gender recognition using thermal images. Based on these results, we propose a recognition method using the MEAN map as the quality measurement of image regions for visible light images, and the STD map as the quality measurement of image regions for thermal images (Method 3). Using Method 3, Equation (3) is changed to Equations (8) and (9) as follows for visible light and thermal images, respectively:

(8) $${\text{wHOG}}_{visible}=\left(MEAN_{ij}\times vHOG_{ij}\right)$$

(9) $${\text{wHOG}}_{thermal}=\left(STD_{ij}\times tHOG_{ij}\right)$$

Although we can directly use the extracted feature for gender recognition using the SVM, the direct use of the feature causes the problems of redundant information and high dimensionality of the feature. In our design, the size of input image is 128 × 64 pixels. The size of block is 16 × 16 pixels with the stride of 8 pixels in horizontal and vertical directions, respectively. Consequently, we have 15 (q=15) and 7 (p=7) sub-blocks in vertical and horizontal directions, respectively, from the input image. For each sub-block, there are 36 histogram bins are extracted. Consequently, a feature vector with 3780 (15 × 7 × 36) histogram bins is extracted for each input image. Using all 3780 histogram bins can cause difficulties for training and testing procedures. In addition, noise is still associated with the extracted features. Therefore, we use principal component analysis (PCA) to reduce the dimensionality and noise associated with the image features. Using PCA, we can reduce the dimension of input features to the SVM by employing a small number of principal components for which the recognition error is smallest. The exact number of principal components is experimentally found during the training phase.

As mentioned in Section 2.1, in our experiments, we will exploit all combinations of methods of combining visible light and thermal images for the recognition problem. First, feature-level fusion is performed by concatenating the wHOG features of visible light and thermal images to form a new feature that contains the gender information from both kinds of images. In the score-level fusion approach, gender is first recognized using individual features from visible light and thermal images using the first SVM layer. The outputs of the first SVM layer are the decision scores that indicate the probability of visible light and thermal images belonging to male or female classes. To combine the two kinds of images, the two scores obtained by the first SVM layer (using visible light and thermal images) are concatenated to form a 2-D feature vector, as shown in Figure 1. Finally, a second SVM layer is used to recognize gender using this 2-D feature vector. In our experiments, two kinds of SVM kernel are used, including the liner kernel and RBF kernel. In addition, in our experiments, we use the OpenCV library (version 2.4.9, Intel Corp., Santa Clara, CA, USA) to implement the PCA and SVM [24].

## 3. Experimental Results

### 3.1. Description of Database, Performance Measurement, and Experimental Setup

There exist open databases which can be used for body-based recognition such as human identity or gender recognition. However, they include only visible light images [25,26,27] or thermal images [28] in the database. Therefore, these databases cannot be used in our research because the dataset including both the visible light and thermal images captured simultaneously is necessary. Although there exists other open database including both visible light and thermal images for pedestrian detection [29], it is difficult to obtain the ground-truth gender information from the images of database because people were captured at very far distance from the cameras. In addition, the number of people in this database is too small (less than 40 persons) to be used for training and testing of gender classification in our research.

**Figure 5:**
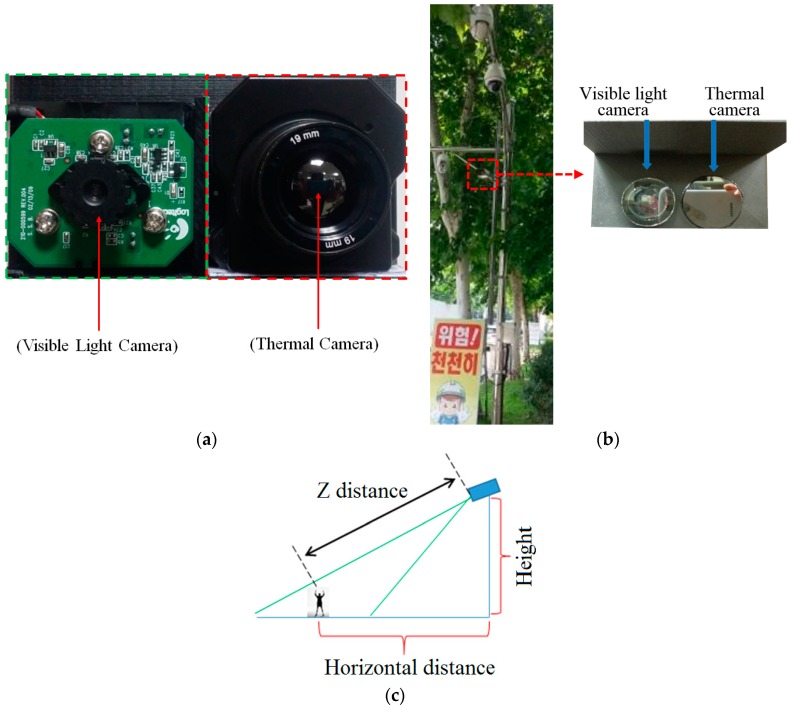
Dual-camera set up that combines visible light and thermal cameras, as used to collect the database in our experiments: (**a**) dual-camera system; (**b**) setup of our camera system in actual surveillance environments; (**c**) distances between camera and user with the height of camera.

In previous research by Nguyen et al., a database of 103 persons, including images of 66 males and 37 females, was used in their experiments [16]. Because there is no open database to be used for the evaluation of our method and in order to test the system with a larger database, we collected a new database of 412 persons with different body-view of subjects such as front, back, and side views. Similar to research by Nguyen et al., we placed the cameras (visible light and thermal cameras) near to each other to create a dual-camera set up, as shown in Figure 5a, and placed it at a height of roughly 6 m. This set up is used as a simulation of a normal surveillance system. Figure 5b shows the example of setup of our system in actual surveillance environments. In our experiments, we used a webcam camera (C600, Logitech, Lausanne, Switzerland) [30] for capturing visible light images, and a Tau2 camera (FLIR systems, Wilsonville, OR, USA) [31] for capturing thermal images. The visible light camera captures images with a size of 800 × 600 pixels. The thermal camera captures images with a size of 640 × 480 pixels. We prevent rain from entering the visible light and thermal cameras by attaching a glass cover (conventional transparent glass and germanium glass transparent to medium-wavelength infra-red (MWIR) and long-wavelength infra-red (LWIR) light [32] for the visible light and thermal cameras, respectively) to the front of each camera, as shown in the right image of Figure 5b.

In our research, we aim at the gender recognition in surveillance system (not retail domain). Gender recognition in surveillance system has a lot of advantages. One of them is that it can enhance the accuracy of face recognition by pre-classification based on gender in 1-to-N matching of face recognition. For example, if the face recognition system should match the input face with 1000 faces (criminal suspicious faces or faces of missing children) in the databases (where the numbers of male are 500) and the gender of people of the input image is classified as male in advance by our system, the face recognition system can match the input face only with 500 male faces (not 1000 faces), which can highly increase the matching speed and accuracies. In addition, the gender information can be helpful for intelligent surveillance system to determine the criminal suspicious situation. For example, in case that a female is persistently traced by a male in alley at night (where there is not other people), it can be determined as criminal suspicious situation. By notifying this situation to the nearest police station, criminal can be prevented. In addition, in some public areas, knowing gender can help the manager create restricted male and female areas [4].

Therefore, in our research, we aim at the gender recognition in surveillance system (not retail domain), and its necessity is increased in intelligent surveillance system. In case that the camera is installed at 3 m in surveillance system, pedestrian can easily break the camera. Therefore, the camera is usually installed at a height of roughly 6 meters or higher in conventional surveillance system. Based on Figure 5c, the height of our camera system and horizontal distance (between the camera system and user) are respectively about 6 m and 11 m. Therefore, Z distance between the camera system and user is about 12.5 m when our dataset was collected.

**Figure 6:**
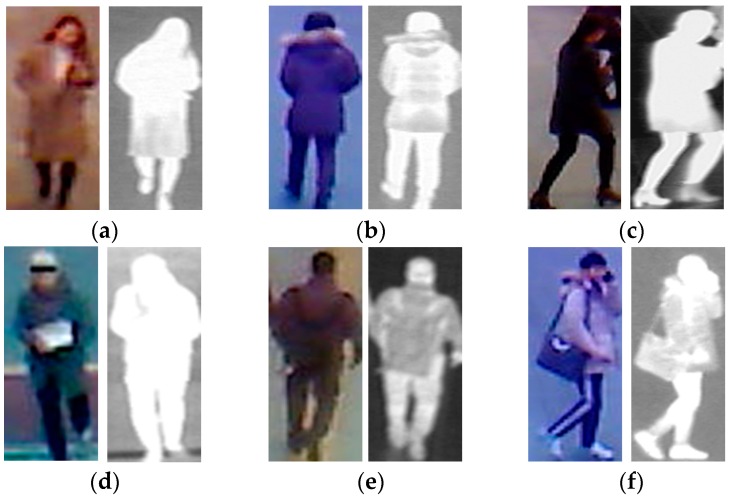
Example of visible light and thermal images in the collected database used in our experiments: (**a**–**c**) visible light-thermal image pairs of the female class with (**a**) front view; (**b**) back view; and (**c**) side view; (**d**–**f**) visible light-thermal image pairs of the male class with (**d**) front view; (**e**) back view; and (**f**) side view.

For each person, 10 images were respectively captured by visible light and thermal cameras to simulate the variation of body shape. Therefore, although some images are captured for the same person, they are still different, because of the capturing conditions, body-pose etc. In total, we collected a database including 8240 images (4120 visible light images and 4120 corresponding thermal images). Among the 412 subjects, there were 254 males and 158 females. Using a larger number of persons, we can measure the more correct recognition accuracy using our proposed method, as compared to previous research by Nguyen et al. [16]. In Table 2, we present a description of the collected database used in our experiments. For visualization purposes, we also show some example images of human bodies in our collected database in Figure 6. As shown in Figure 6, our database contains images of the human body with large variations in body-view (front, back, and side view), background, clothes, and accessories, etc. In addition, we make our all database (used in our research) [33] available for others to use in their own evaluations, from which comparisons with our method on same database can be done.

To measure the recognition performance of our proposed system, we randomly divided the collected database in Table 2 into learning and testing sub-databases five times to perform a five-fold cross-validation method. Consequently, we obtained five learning sub-databases, each sub-database contain images of 204 males and 127 females; and the five testing sub-databases, each sub-database contains images of 50 males and 31 females. Using the learning sub-databases, we learn the parameters of the SVM kernel that best classifies the male and female classes. With the learnt parameters and the SVM kernel, we evaluate the recognition performance using testing sub-databases.

Table 3 shows a detailed description of the learning and testing sub-databases in our experiments.

For the recognition problem, the equal error rate (EER) is normally used to measure the recognition accuracy of the recognition systems. The EER indicates errors for which the false acceptance rate (FAR) is equal to the false rejection rate (FRR). In our problem of gender recognition, we have two classes: male and female. If we define male as “class 1” (genuine class) and female as “class 2” (importer class), then the FAR is the error by which an image of a female is falsely recognized as a male image. Conversely, the FRR indicates the error by which an image of a male is falsely recognized as a female image. By definition, systems with a small value of EER are systems with high recognition performance; whereas systems with the larger values of EER indicate systems with poor recognition performance. In our experiments, EER is measured using five learning and testing sub-databases, and the final EER of the system is measured as the average of the five results from five learning and testing sub-databases.

### 3.2. Gender Recognition Using Our Proposed Method

To compare the recognition accuracy of our proposed method with that of the previous method by Nguyen et al. [16], we first perform experiments using the method of Nguyen et al., using our collected database in Table 2 and Table 3. The previous method by Nguyen et al., directly uses the HOG feature extracted from the visible light and thermal images for gender recognition using the SVM, without considering the quality measurement of local regions of input images. Therefore, their method corresponds to our proposed method by removing the quality measurement method in Figure 1. Similar to experiments in their paper, we measured the recognition accuracies of systems using both feature-level fusion and score-level fusion methods using two kinds of SVM kernels, i.e., the linear kernel and the RBF kernel. The details of our experimental results are shown in Table 4 and Table 5. In Table 4, we show the recognition results of systems that only use a single kind of image (only visible light images or only thermal images) in the recognition task. Similar to this table but for combining the visible light and thermal images, Table 5 shows the recognition results of feature-level fusion and score-level fusion methods. In Table 4, Table 5, Table 6, Table 7, Table 8, Table 9 and Table 10, the GAR implies the genuine acceptance rate and it is calculated as (100-FRR)(%). In these tables, FAR and GAR at the EER point are shown in bold type.

**Figure 7:**
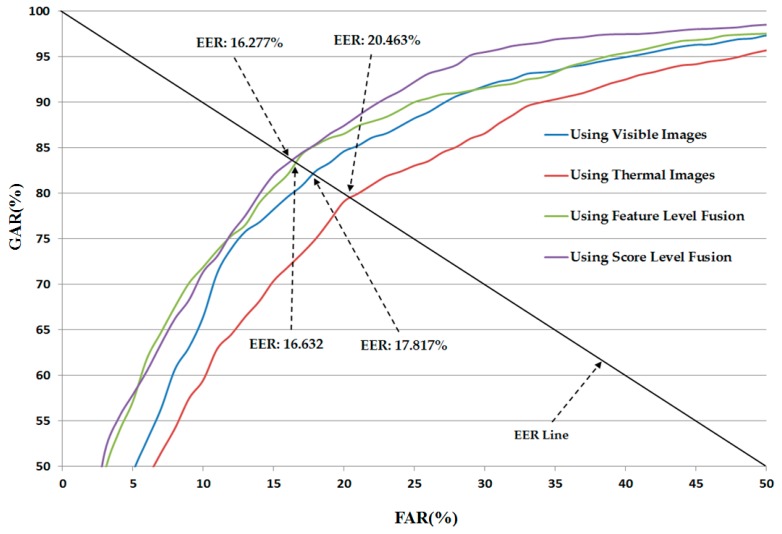
The average ROC curve of a previous recognition method [16] with different kinds of images and combination methods.

In Figure 7, we show the average receiver operating curve (ROC curve) of the previous recognition system using our collected database. In detail, the best recognition accuracy was obtained with an EER of 16.277% using the score-level fusion method and RBF kernel in both layers of the SVM classifier. This result is much smaller than the recognition accuracy when we use only visible light images (17.817%) or only thermal images (20.463%), or the feature-level fusion method (16.632%). As shown in Table 4 to Table 5 and Figure 7, we confirm that the combination of visible light and thermal images can help enhance the recognition accuracy of the recognition system. In addition, the RBF kernel is superior to the linear kernel for the recognition problem. However, the recognition accuracy is slightly worse than the previous results reported by Nguyen et al. [16]. These results are caused by the differences of the databases. In previous research, Nguyen et al., used a database of 103 persons, which is much smaller than the 412 persons of our database. Using a larger database, our experiment can reflect more correct recognition accuracies.

As explained in Section 2, we proposed the use of two quality measurement methods of image regions for wHOG feature formation, including the MEAN map and the STD map. To evaluate the efficency of each quality measurement method (the MEAN map and the STD map), we measure the recognition accuracies of the recognition system using each individual quality measurement method (Method 1 for use with the MEAN map and Method 2 for use with the STD map as the quality assessment of image regions for both visible light and thermal images) in our next experiments.

In the second experiment, we use the MEAN map as the quality measurement of image regions for both visible light and thermal images (Method 1). Using Equations (4) and (5), we extract the wHOG feature for gender recognition by combining the HOG feature and the MEAN map of sub-blocks of the input thermal images. With the extracted weighted HOG feature, we perform gender recognition using the SVM with two kinds of SVM kernels, i.e., the linear kernel and the RBF kernel. In Table 6, we show the recognition accuracy of the recognition system in this experiment. As shown in Table 6, the best recognition accuracy when using only visible light images was obtained with an EER of 15.219% using the RBF kernel of the SVM; the best recognition accuracy when using only thermal images was obtained with an EER of 18.335% using the RBF kernel. Compared to recognition accuracies of the system that does not consider the quality measurement of image regions in Table 4, we see that the recognition accuracies (EER) were reduced from 17.817% to 15.219% when using only visible light images; and from 20.463% to 18.335% when using only thermal images for the cases without and with consideration of the quality measurement of image regions, respectively. These results indicate that the MEAN map can enhance the recognition accuracies of the gender recognition system. 

Similar to the second experiment for Method 1, we performed our third experiment using Method 2, which uses the STD map of the input thermal images as the quality measurement of local image regions for constructing the wHOG feature. For these experiments, the wHOG features are obtained using Equations (6) and (7) in Section 2. The detailed experimental results are shown in Table 7. As shown in this table, the use of STD map helps to reduce the recognition error from 17.817% (for the case without consideration of the quality measurement of the image’s local regions) to 16.669% using only visible light images for the recognition task. When using only the thermal images, the use of the STD map can also reduce the recognition error by producing an EER of 18.257% which is smaller than the error of 20.463% (in Table 4) produced by the system that does not consider the quality measurement of local image regions, and 18.335% produced by the system that uses the MEAN map of the thermal images as its quality measurement of local image regions.

As shown in Table 6 and Table 7, we obtained the best recognition results of 15.219% and 18.257% using only visible light images and only thermal images for the recognition task, respectively. These EERs values are much smaller than the EERs of 17.817% and 20.463% of the system that does not consider the qualities of image regions using visible light and thermal images, respectively. These results were obtained using the MEAN map on visible light images and the STD map on thermal images and the RBF kernel of the SVM. Based on these results, we can find that the use of quality measurement for image regions can enhance the recognition accuracy of the gender recognition system. In addition, we can see that the MEAN map is more efficient for quality measurement of the image’s local regions for visible light images than the STD map; the STD map is more efficient as the quality measurement of the image’s local regions for thermal images than the MEAN map. Therefore, we propose the use of the MEAN map for quality measurement of local regions for visible light images and the STD map as the quality measurement of local regions for the thermal images.

Based on the experimental results of our second and third experiments (Methods 1 and 2), we perform our fourth experiments using our proposed gender recognition method in which the MEAN map is used as the quality measurement of local regions of visible light images and the STD map is used as the quality measurement of local regions of thermal images. Similar to the first experiment, we perform gender recognition using both feature-level fusion and score-level fusion with two kinds of SVM kernel of linear and RBF kernels. In this experiment, the wHOG features of visible light images are obtained using Equation (8) and the wHOG feature of thermal images are obtained using Equation (9) by combining the MEAN map and the STD map with the HOG features of visible light and thermal images, respectively. The detail recognition accuracies of these experiments are shown in Table 8 and Table 9. Table 8 shows the experimental results when we used only visible light or only thermal images for gender recognition, whereas Table 9 shows the recognition results by combining the visible light and thermal images.

**Figure 8:**
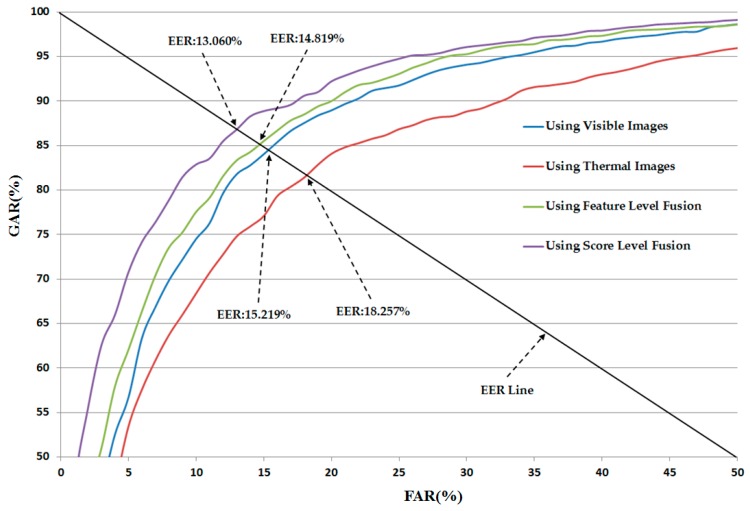
Average ROC curve of our proposed method for gender recognition using different kinds of images and combination methods.

As shown in Table 9, the feature-level fusion method produced a recognition error (EER) of 16.162% and 14.819% using the linear and RBF kernels of the SVM, respectively. These errors are much smaller than the errors of 17.892% and 16.632% of the system that does not consider the qualities of local regions of images using the linear and RBF kernels of the SVM (see Table 5), respectively. Using score-level fusion, the best recognition accuracy was obtained with an EER of 13.060% using the the RBF kernel in both SVM layers. This result is also much smaller than that of 16.277% for the system that does not consider the quality of local regions of input images (see Table 5). In addition, this result (an EER of 13.060%) is also the best recognition accuracy among those of the other methods in Table 4, Table 5, Table 6, Table 7, Table 8 and Table 9. Based on these results, we can find that the proposed method can enhance the recognition accuracy of gender recognition using visible light and thermal images of the human body. Figure 8 shows the ROC curve of our proposed method using our collected database.

In Table 10, we summarize the recognition results of our experiments. This table shows that our proposed method produces much better recognition accuracies, as compared to the accuracies obtained using the previous method.

Although the Table 10 gave the difference between performances of difference configurations of our proposed system and the previous sytem, we further performed experiemnts to verify the differences statistically. For this purpose, we measured the differences of performances (recognition accuracies) of various configurations of recognition systems using the *t*-test method [34]. The *t*-test method is statistical tool that is usually used to verify the difference between the mean values of two independent random variables. The details of experimental results are shown in Table 11 and Table 12. In Table 11, we showed the *t*-test results to verify the difference of performance of various system configurations using our proposed feature extraction method, including the system using single visible light images; system using single thermal images; system using the feature level fusion of visible light and thermal images; and the system using the score level fusion of visible light and thermal images. As shown in Table 11, the *p*-value of the system using only visible light images and the system using only thermal images was about 0.025929, which is smaller than 95% (0.05) significant level. Therefore, the null hypothesis for the *t*-test, that there is no difference between the performances of system using only visible light images and system using only thermal images for gender recognition, may be rejected at the significant level of 95%. The *p*-value of system using only visible light images and the system using score level fusion of visible light and thermal images was about 0.004782, which is smaller than 99% (0.01) significant level. The *p*-value of system using only thermal images and the system using score level fusion of visible light and thermal images was about 0.001461, which is also smaller than 99% (0.01) significant level. Through these results, we can find that the performances of system using only visible light images or system using only thermal images are statistically different from the performance of the system using score level fusion at the significant level of 99%. In addition, the *p*-value of the system using feature level fusion and the system using score level fusion was about 0.063061. This *p*-value is smaller than 93% (0.07) significant level. Therefore, we can find that there is a statistical difference between the performances of system using score level fusion and the system using feature level fusion at the significant level of 93%.

In Table 12, we showed the *t*-test results to verify the difference of performance of various system configuration using our proposed method and previous method [16]. For this purpose, we measured the *p*-values of two random variables, including the performance of system using previous method [16] and the performance of system using our proposed method. There are four different configurations were used, including system using only visible light images, system using only thermal images, system using the combination of visible light and thermal image based on feature level fusion approach, and the system using the combination of visible light and thermal images based on the score level fusion approach. As shown in this table, the *p*-values between our method and previous one [16] using only visible light image was about 0.039456. This value is smaller than 95% (0.05) significant level. Therefore, the performances of ours and previous system [16] that use only visible light images for gender recognition are statistically different at the significant level of 95%. Similarly, we find that the performances of systems that use only thermal images for gender recognition are statistically different between ours and previous method at the significant level of 95% (*p*-value of 0.025682); performances of systems that use feature level fusion of visible light and thermal images for gender recognition are statistically different between these two methods at the significant level of 99% (*p*-value of 0.002941); and performances of systems that use score level fusion of visible light and thermal images for gender recognition are statistically different between these two methods at the significant level of 99% (*p*-value of 0.00508).

**Figure 9:**
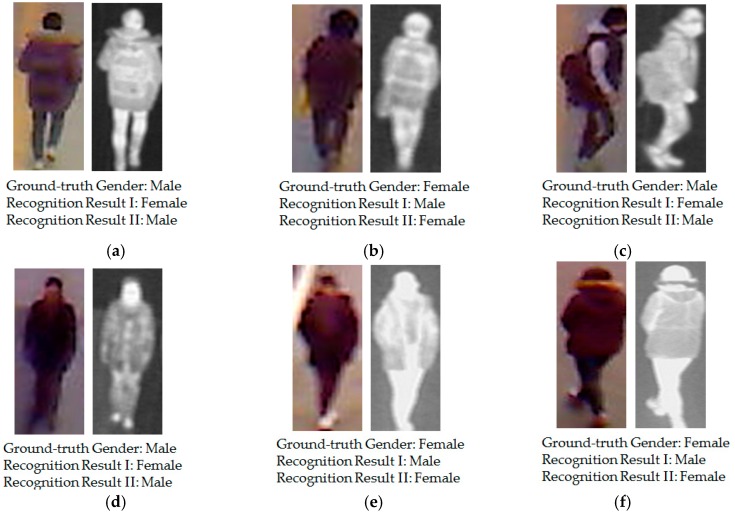
Example recognition results for our proposed method, as compared to the previous recognition method: (**a**) male image in the back view; (**b**) and (**f**) female images in the back view; (**c**) male image in the side view; (**d**) male image in the front view; and (**e**) female image in the front view.

For demonstration purposes, we show some examples of recognition results of the previous recognition sytem [16] and compare them to those of our proposed method in Figure 9. In this figure, “Recognition Result I” indicates the recognition result when using the previous method by Nguyen et al. [16], and “Recognition Result II” indicates the recognition result when using our proposed method. These examples show that although the privous method produced incorrect recognition results (from male to female and vice versa), the proposed methodcorrectly recognizes gender from the human in input images. 

**Figure 10:**
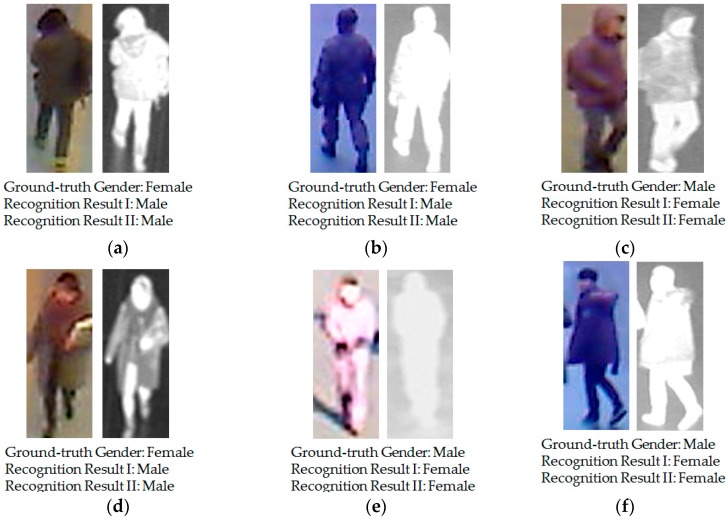
Example of recognition results of our proposed method where the error cases were occurred: (**a**,**b**) female images in back view; (**c**,**f**) male images in side view; (**d**) female image in front view; and (**e**) male image in front view.

Although our proposed method was demonstrated to enhance the recognition accuracy of the recognition system, it still has errors (at roughly 13%, as shown in Table 9 and Table 10). In Figure 10, we show several error cases produced by our proposed method. Similar to Figure 9, “Recognition Result I” indicates the recognition result when using the previous method by Nguyen et al. [16]; and “Recognition Result II” indicate the recognition result when using our proposed method. In Figure 10a,b, it is difficult to distinguish if the persons in the two images are females, even by human perception. The person in Figure 10b is wearing an army uniform and the person in Figure 10a has short hair as viewed from the back. These conditions makes the recognition system produce incorrect recognition results. A similar situation occurs in Figure 10c,d. In Figure 10e,f, although we can recognize the gender of persons in these images using human perception, the unusual body pose (in Figure 10f) and the poor capturing conditions (in Figure 10e cause our proposed system to produce the incorrect recognition results. As shown in this figure, the corresponding recognition results using the previous method by Nguyen et al., are also wrong for these input image cases. These results are caused by the very large variation of images of the human body such as the variation of wearing clothes, wearing accessories, the capturing view, etc. In the future, we will continue to study these kinds of negative effects so as to enhance the recognition accuracy.

We included the comparative experiments with a similar feature extraction method, called entropy weighted histograms of oriented gradients (EWHOG) that was proposed by Liu et al. [35]. The EWHOG is a new method that enhances the power of traditional HOG feature by measuring the amount of information contained in the blocks of image using the entropy of the HOG feature extracted in each block. The entropy reflects the degree of uncertainty of texture feature in image’s blocks. Therefore, the blocks with large variation of texture should have larger entropy values than those that have small variation (plain texture). 

The detailed experimental results are shown in Table 13. As shown in this table, the best recognition accuracies by the EWHOG method [35] were obtained with EER of about 14.135%. In addition, the use of both visible light and thermal images showed the better recognition accuracies than the use of single visible light or single thermal image for gender recognition by the EWHOG method. Compared to recognition accuracy obtained by our method in Table 10 and Table 13, we can see that our method outperforms the EWHOG method by producing the lower error than the EWHOG method (13.060% vs. 14.135%). 

**Figure 11:**
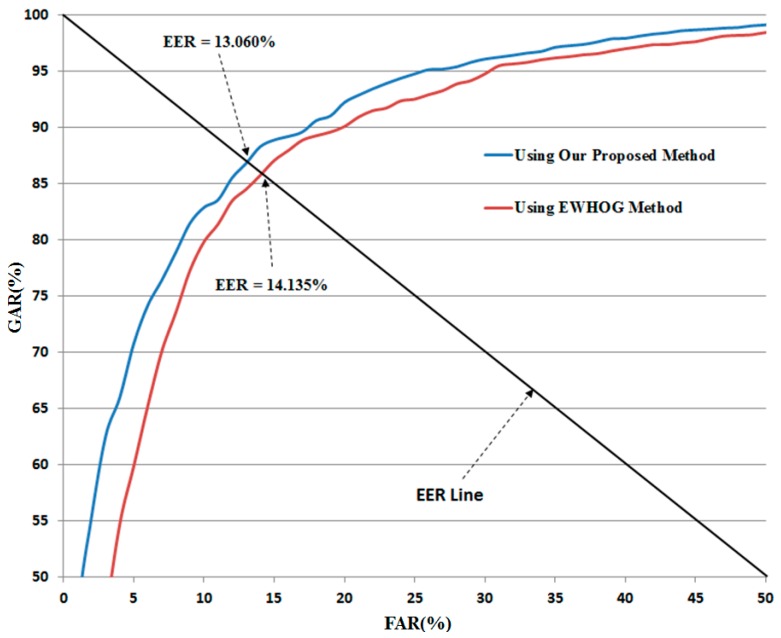
Average ROC curve of our method and EWHOG method for gender recognition.

In Figure 11, we show the ROC curve of recognition system using our method and the EWHOG method, which shows that the recognition accuracy by our method is higher than that by the EWHOG method.

As a next experiment, we measured the processing time of our proposed method. For this purpose, we used a desktop computer with an Intel Core i7 CPU (3.5 GHz, Intel Corp., Santa Clara, CA, USA) with 8 GB of RAM memory. The recognition program was written using the C++ programming language. The detailed experimental results are shown in Table 14. In conclusion, our proposed method can execute at the speed of 36.14 frames per second (1000/27.6679). In order to run our algorithm on the camera system having very low processor or on the server to process dozen of streams in parallel, the processing speed by our method should be enhanced. However, as shown in Table 14, most processing time was taken at the stage of human body detection (this is not main part of our research because our research is mainly focused on gender recognition). 

As future works, we would research about the method of enhancing the processing speed of human body detection, by which our gender recognition method can be operated on the camera system having very low processor or on the server to process dozen of streams in parallel.

We performed additional experiments to measure the recognition accuracies of our system with and without applying the PCA. The detailed experimental results were shown in Table 15. As shown in these experimental results, the use of PCA helped enhancing the recognition accuracy about 2% compared to the case of without applying the PCA using our proposed method (13.060% vs. 15.072%). 

In addition, the use of PCA can also help reducing the redundant features. Originally, we extracted a feature vector of 3780 components (a feature vector in 3780-dimensional space) for a visible light or thermal image using our proposed weighted-HOG method. Consequently, the recognition system must process a vector in 7560-dimensional space (3780 × 2) for a combination of visible light and thermal images using the feature level fusion approach; or processes two vectors in 3780-dimensional space separately using the score level fusion approach. Processing with high dimensional feature vector not only increases processing time of recognition system, but also requires more memory for storing feature vector. Therefore, the PCA is deserved in our proposed method.

The thermal image has a special characteristic that the appearance of objects on the thermal image is only dependent on the object’s temperature. While the temperature of human body is normally a constant of about 37°, the temperature of background is strongly dependent on the environment temperature and normally lower than that of human body’s temperature. Consequently, the background regions appear darker (lower pixel value) than those of human body regions in the thermal image as shown in Figure 6, Figure 9 and Figure 10. Based on this characteristic, although the background regions can have very complex structure or uneven intensity, they are displayed darker than the body regions in thermal image, and the corresponding mean values of sub-blocks of background are lower than those of human body. Therefore, we use the mean value of sub-blocks of thermal image to evaluate the amount of body-based gender information contained in the corresponding block in the visible light image with an assumption that the misalignment of visible light and thermal image of human body is small. Using this method, we can assign low weight values to background regions even though they contain very complex structure or uneven intensity.

The background regions appear darker than those of human body regions in the thermal image, and the difference among pixel values inside human body is not large in the thermal image as shown in Figure 6, Figure 9 and Figure 10. Therefore, the sub-blocks from the boundary between human body and background usually show higher standard deviation than those from background and inside of human body in the thermal image. Based on this characteristic, we use the standard deviation as the quality assessment of blocks of thermal image. Using the standard deviation measurement, the background blocks and the blocks which belong to inner part of human body have small weight values, while the blocks which belong to the boundary of human body have larger weight values even though the background regions contain very complex structure or uneven intensity. Consequently, we can obtain the body-based gender information from the shape of human body in the thermal images.

## 4. Conclusions

In this paper, we have proposed a new gender recognition method using images of the human body. To overcome the limitations of previous researches that use only visible light images for the recognition task, we utilize both visible light and thermal images of the human body. The use of thermal images in addition to the visible light images not only enhances the recognition results, but also enhances both the human detection result and background removal. Because the background regions do not contain gender information, we proposed a quality measurement method to reduce the effects of background regions on recognition performance. Based on the characteristics of thermal images, we propose a quality measurement method for local regions in the human body images using the mean and standard deviation of image sub-blocks of thermal images. With the HOG feature and quality measurement extracted from visible light and thermal images, we constructed a new image feature, called wHOG, for recognition purposes. The experimental results showed that the recognition accuracies by our method were higher than previous method for gender recognition.

In future work, so as to enhance the recognition accuracy, we will continue to investigate the negative effects influencing the gender recognition system, such as the effects of body pose (front view, back view, side view), illumination condition, and capture distance. 

## Figures and Tables

**Table 1:** Summary of previous studies on image-based gender recognition.

Categories		Strength	Weakness
Using face images [5,6,7,8,9]		- Very high recognition rate.	- Requires the cooperation of subjects to obtain the face image; not suitable for surveillance system applications. - Difficult to recognize the gender of very young people.
Using gait or a 3D model of the human body [10,11,12,13]		- Good estimation result can be obtained by analyzing the 3D body shape of a human or a sequence of gait images.	- Requires a sequence of images to obtain average gait images [10,11]. - Requires the cooperation of subjects to obtain a good estimation result. In addition, the capturing device is expensive, as it uses a laser scanner [12,13].
Using 2D images of the human body	Using visible light images [14,15]	- Recognition is performed using images of the human body. - Does not require the cooperation of the subject. Therefore, this method is suitable for surveillance systems in shopping malls, airports, etc.	- Recognition accuracy is limited because only visible light images are used. - Strong effects of background, body pose, cloth variation, etc.
	Using visible light and thermal images without quality assessment [16]	- Higher recognition accuracy is obtained by utilizing gender information in both visible light and thermal images of the human body. - Does not require the cooperation of the subject. Therefore, this method is suitable for surveillance systems in shopping malls, airports, etc.	- Recognition accuracy is still affected by the background, variations of body pose, random clothing. - More expensive than a system that uses only visible light images because an additional thermal camera sensor is needed.
	Using visible light and thermal images with quality assessment (the proposed method)	- Better recognition accuracy is obtained by utilizing gender information in both visible light and thermal images of the human body based on quality assessment. - Does not require the cooperation of the subject. Therefore, this method is suitable for surveillance systems in shopping malls, airports, etc. - Effects of background regions on recognition accuracy are significantly reduced by the quality assessment method.	- More expensive than a system that only uses visible light images because an additional thermal camera sensor is needed. - Still has the negative effects of the difference of body-pose, clothes, accessories, etc.

**Table 2:** Description of the collected database for our experiments (10 visible light images/person, and 10 thermal images/person).

Database	Males	Females	Total
Number of Persons	254	158	412 (persons)

**Table 3:** Detailed description of learning and testing sub-databases used in our experiments.

Database		Males	Females	Total
Learning Database	Number of Persons	204 (persons)	127 (persons)	331 (persons)
	Number of Images	4080	2540	6620 images
		(204 × 20 images)	(127 × 20 images)	
Testing Database	Number of Persons	50 (persons)	31 (persons)	81 (persons)
	Number of Images	1000	620	1620 images
		(50 × 20 images)	(31 × 20 images)	

**Table 4:** Recognition accuracies of previous method [16] using single visible light image or single thermal image for gender recognition (unit: %).

Feature Extraction Method	SVM Kernel	Accuracies of Recognition System Using Single Kind of Images					
		Using Only Visible Light Images			Using Only Thermal Images		
		EER	FAR	GAR	EER	FAR	GAR
HOG	Linear	23.962	15.00	64.14	25.360	20.00	65.20
			20.00	71.92		25.00	74.02
			**23.98**	**76.057**		**25.360**	**74.641**
			25.00	77.36		30.00	79.36
			30.00	81.72		35.00	82.56
	RBF	**17.817**	10.00	66.44	**20.463**	15.00	70.37
			15.00	78.22		20.00	79.08
			**17.820**	**82.186**		**20.480**	**79.554**
			20.00	84.60		25.00	83.02
			25.00	88.22		30.00	86.56

**Table 5:** Recognition accuracies of previous method [16] using the combination of visible light and thermal images with feature-level fusion and score-level fusion approaches (unit: %).

Feature Extraction Method	First SVM Layer Kernel	Accuracy of Recognition System Using Combined Images						
		Feature-Level Fusion			Score-Level Fusion			
		EER	FAR	GAR	Second SVM Layer Kernel	Accuracy		
						EER	FAR	GAR
HOG	Linear	17.892	10.00	70.80	Linear	19.955	10.00	63.40
							15.00	71.08
			15.00	78.70			**19.960**	**80.050**
							20.00	80.08
			**17.90**	**82.116**			25.00	84.96
					RBF	20.059	15.00	70.385
			20.00	84.32			20.00.	79.841
							**20.060**	**79.941**
			25.00	86.947			25.00	85.259
							30.00	88.901
	RBF	**16.632**	10.00	71.90	Linear	16.333	10.00	71.46
							15.00	81.80
			15.00	80.59			**16.340**	**83.675**
							20.00	87.64
			**16.660**	**83.396**			25.00	92.32
					**RBF**	**16.277**	10.00	71.368
			20.00	86.52			15.00	81.99
							**16.280**	**83.726**
			25.00	90.00			20.00	87.408
							25.00	92.21

**Table 6:** Recognition accuracies of our proposed method using the MEAN map for the quality assessment of image regions for both visible light and thermal images (unit: %).

wHOG Method	SVM Kernel	Recognition Accuracies					
		Using Only Visible Light Images			Using Only Thermal Images		
		EER	FAR	GAR	EER	FAR	GAR
Using MEAN Map (**Method 1**)	Linear	20.476	15.00	72.42	23.098	15.00	65.92
			20.00	79.20		20.00	73.60
			**20.480**	**79.527**		**23.120**	**76.924**
			25.00	83.82		25.00	78.90
			30.00	87.56		30.00	82.58
	**RBF**	**15.219**	10.00	74.52	**18.335**	10.00	66.65
			15.00	84.04		15.00	76.56
			**15.220**	**84.782**		**18.340**	**81.670**
			20.00	88.96		20.00	83.94
			25.00	91.76		25.00	86.79

**Table 7:** Recognition accuracies of our proposed method using the STD map for quality assessment of image’s regions for both visible light and thermal images (unit: %).

Quality Measurement Method	SVM Kernel	Recognition Accuracies					
		Using Only Visible Light Images			Using Only Thermal Images		
		EER	FAR	GAR	EER	FAR	GAR
Using STD Map **(Method 2)**	Linear	20.962	15.00	70.94	22.410	15.00	65.58
			20.00	78.68		20.00	74.52
			**20.980**	**79.055**		**22.420**	**77.601**
			25.00	82.92		25.00	79.26
			30.00	86.32		30.00	82.96
	**RBF**	16.669	10.00	70.68	**18.257**	10.00	68.40
			15.00	81.30		15.00	77.09
			**16.680**	**83.342**		**18.260**	**81.747**
			20.00	86.62		20.00	84.08
			25.00	90.00		25.00	86.85

**Table 8:** Recognition accuracies of our proposed method (using the MEAN map for quality assessment of image regions of visible light images and the STD map for quality assessment of image regions of thermal images) using single kind of images (unit: %).

wHOG Method	The First SVM Layer Kernel	Accuracy of Recognition System Using Single Kind of Images					
		Using Only Visible Light Images			Using Only Thermal Images		
		EER	FAR	GAR	EER	FAR	GAR
Using MEAN and STD Maps **(Method 3)**	Linear	20.476	15.00	72.42	22.410	15.00	65.58
			20.00	79.20		20.00	74.52
			**20.480**	**79.527**		**22.420**	**77.601**
			25.00	83.82		25.00	79.26
			30.00	87.56		30.00	82.96
	RBF	15.219	10.00	74.52	**18.257**	10.00	68.40
			15.00	84.04		15.00	77.09
			**15.220**	**84.782**		**18.260**	**81.747**
			20.00	88.96		20.00	84.08
			25.00	91.76		25.00	86.85

**Table 9:** Recognition accuracies of our proposed method (using MEAN map for quality assessment of image regions of visible light images and the STD map for quality assessment of image regions of thermal images) using feature-level fusion and score-level fusion approaches (unit: %).

wHOG Method	The First SVM Layer Kernel	Accuracy of Recognition System Using Combined Images						
		Feature-level Fusion			Score-level Fusion			
		EER	FAR	GAR	The Second SVM Layer Kernel	Accuracy		
						EER	FAR	GAR
Using MEAN and STD Maps **(Method 3)**	Linear	16.162	10.00	71.96	Linear	16.197	10.00	73.16
							15.00	82.32
			15.00	82.66			**16.220**	**83.827**
							20.00	86.98
			**16.180**	**83.857**			25.00	90.22
					RBF	16.595	10.00	71.726
			20.00	87.54			15.00	81.435
							**16.60**	**83.410**
			25.00	90.68			20.00	86.231
							25.00	90.027
	RBF	**14.819**	5.00	62.08	Linear	13.452	5.00	70.60
							10.00	82.00
			10.00	77.56			**13.460**	**86.556**
							15.00	88.82
			**14.840**	**85.203**			20.00	92.16
					**RBF**	**13.060**	5.00	70.79
			15.00	85.52			10.00	82.88
							**13.080**	**86.960**
			20.00	90.00			15.00	88.88
							20.00	92.22

**Table 10:** Summary of recognition accuracy using our proposed method and the previous method (unit: %).

Method	Using Single Visible Light Images			Using Single Thermal Images			Feature-Level Fusion			Score-Level Fusion		
	EER	FAR	GAR	EER	FAR	GAR	EER	FAR	GAR	EER	FAR	GAR
Previous Method [16]	17.817	10.00	66.44	20.463	15.00	70.37	16.632	10.00	71.90	**16.277**	10.00	71.368
		15.00	78.22		20.00	79.08		15.00	80.59		15.00	81.99
		**17.820**	**82.186**		**20.480**	**79.554**		16.660	83.396		**16.280**	**83.726**
		20.00	84.60		25.00	83.02		20.00	86.52		20.00	87.408
		25.00	88.22		30.00	86.56		25.00	90.00		25.00	92.21
Our Method	15.219	10.00	74.52	18.257	10.00	68.40	14.819	5.00	62.08	**13.060**	5.00	70.79
		15.00	84.04		15.00	77.09		10.00	77.56		10.00	82.88
		**15.220**	**84.782**		**18.260**	**81.747**		**14.840**	**85.203**		**13.080**	**86.960**
		20.00	88.96		20.00	84.08		15.00	85.52		15.00	88.88
		25.00	91.76		25.00	86.85		20.00	90.00		20.00	92.22

**Table 11:** The *p*-value of performances of different system’s configurations using our proposed method (N/A: Not available).

Method	Using Only Visible Light Images	Using Only Thermal Images	Feature Level Fusion	Score Level Fusion
Using Only Visible Light Images	N/A	0.025929	0.113365	0.004782
Using Only Thermal Images	0.025929	N/A	0.009277	0.001461
Feature Level Fusion	0.113365	0.009277	N/A	0.063061
Score Level Fusion	0.004782	0.001461	0.063061	N/A

**Table 12:** The *p*-value of the performances of different system’s configurations using our proposed method and previous method [16].

Method	Using Only Visible Light Images	Using Only Thermal Images	Feature Level Fusion	Score Level Fusion
*p*-value	0.039456	0.025682	0.002941	0.005080

**Table 13:** Recognition accuracy using EWHOG method (unit: %).

Method	Using Single Visible Light Images			Using Single Thermal Images			Feature-Level Fusion			Score-Level Fusion		
	EER	FAR	GAR	EER	FAR	GAR	EER	FAR	GAR	EER	FAR	GAR
Our Method	15.219	10.00	74.52	18.257	10.00	68.40	14.819	5.00	62.08	**13.060**	5.00	70.79
		15.00	84.04		15.00	77.09		10.00	77.56		10.00	82.88
		**15.220**	**84.782**		**18.260**	**81.747**		**14.840**	**85.203**		**13.080**	**86.960**
		20.00	88.96		20.00	84.08		15.00	85.52		15.00	88.88
		25.00	91.76		25.00	86.85		20.00	90.00		20.00	92.22
EWHOG Method [35]	15.113	10.00	74.840	19.198	10.00	60.880	14.767	5.00	62.200	**14.135**	5.00	59.900
		15.00	84.820		15.00	74.280		10.00	77.300		10.00	79.820
		**15.120**	**84.894**		**19.200**	**80.805**		**14.780**	**85.245**		**14.140**	**85.870**
		20.00	89.213		20.00	81.460		15.00	85.350		15.00	87.060
		25.00	92.270		25.00	84.600		20.00	88.840		20.00	90.080

**Table 14:** The processing time of our proposed method (unit: ms).

Human Body Detection	Quality Measurement	HOG Feature Extraction	Feature Dimension Reduction by PCA	SVM Classification (Layer 1 and 2)	Total
23.130	0.0731	1.6335	2.7548	0.0765	**27.6679**

**Table 15:** The recognition accuracy (EER, %) of recognition system with and without applying PCA.

Method	Using Single Visible Light Images	Using Single Thermal Images	Feature Level Fusion	Score Level Fusion
Without PCA	17.228	20.000	16.126	15.072
With PCA	15.219	18.257	14.819	**13.060**
